# New Codeism in Alabama

**Published:** 1888-04

**Authors:** 


### Editorial.

### NEW CODEISM IN ALABAMA.

The communication of Dr. Davis, of Birmingham, on New Codeism in Alabama, while of peculiar interest to the profession of that State, is not without value in other localities. As expressed in a former editorial in THE JOURNAL, it is highly desirable that there should be judicious and as nearly as possible uniform legislation in all of the States regulating the practice of medicine. Whether the bold attempt in this direction now being made in Alabama is the best and most practicable that can now be devised is a point on which we are not prepared to give an opinion. Indeed, physicians outside of Alabama are too unfamiliar with the requirements of the law and of its practical workings to form one. The subject is, however, so important that we earnestly invite the officers of the State Medical Association of Alabama, or others competent to do so, to furnish us with a synopsis or exposition of the law as it exists and a reply to such objections, if any, as have been made to it. In all the States like evils exist, and if the profession in Alabama have found the best or an effectual remedy for them, if it is not "patented" other States could adopt or imitate it.

